# Combined Effects of Green Tea Extracts, Green Tea Polyphenols or Epigallocatechin Gallate with Acarbose on Inhibition against α-Amylase and α-Glucosidase *in Vitro*

**DOI:** 10.3390/molecules180911614

**Published:** 2013-09-18

**Authors:** Junjie Gao, Ping Xu, Yuefei Wang, Yiqi Wang, Danielle Hochstetter

**Affiliations:** 1 Department of Tea Science, Zhejiang University, 866 Yuhangtang Road, Hangzhou 310058, Zhejiang, China; E-Mails: 21116083@zju.edu.cn (J.G.); zdcy@zju.edu.cn (Y.W.); harmonicminor@hotmail.com (D.H.); 2 Key Laboratory of Horticultural Plant Growth, Development and Quality Improvement, Chinese Ministry of Agriculture, Hangzhou 310029, Zhejiang, China; 3 College of Pharmacy, Zhejiang Chinese Medical University, Hangzhou 310053, Zhejiang, China; E-Mail: yiqitea@126.com

**Keywords:** green tea extracts, polyphenols, EGCG, combination

## Abstract

Green tea, green tea polyphenols and epigallocatechin gallate (EGCG) are confirmed to have beneficial effects in the treatment of diabetes mellitus, and a possible mechanism can be ascribed to their inhibitory effect against α-amylase and α-glucosidase in the digestive tract. In this paper, we first investigated the combined inhibitory effect of green tea extracts, green tea polyphenols or EGCG with acarbose on α-amylase and α-glucosidase *in vitro*. Our results indicated that the interaction between green tea extracts (green tea polyphenols or EGCG) and acarbose was complicated. The combination of green tea extracts, green tea polyphenols or EGCG with acarbose had a synergistic effect on α-amylase and α-glucosidase at low concentrations and the combined effect turned out to be antagonistic at high concentrations according to the Combination Index (CI) values. These findings not only provided some significant quantitative values, but also provide some valuable implications for the combined use of acarbose and GTE (GTP or EGCG) in the treatment of diabetes mellitus.

## 1. Introduction

Diabetes mellitus is a seriously chronic disease accompanied by hyperglycemia which results in the disturbance of the metabolism and brings about attendant diabetic complications such as hypertension, cardiovascular disease and diabetic neuropathy [1]. There is an urgent need for the treatment of diabetes owning to the huge adverse effects induced by diabetes and the fact that the last decade has seen a worldwide explosive increase in diabetes mellitus, especially type 2 diabetes mellitus. It is currently believed that controlling postprandial hyperglycemia is an efficient therapeutic approach to manage diabetes and this process is generally realized by retarding two key enzymes in the digestive system linked to the adsorption of glucose (α-amylase and α-glucosidase), so the search for excellent inhibitors against α-amylase and α-glucosidase has already become a focus of research. 

Recent studies indicate that natural bioactive compounds from dietary fruits and plants such as colored grains [2], sorghum [3], strawberry and raspberry [4] possess inhibitory activity against α-amylase and α-glucosidase and thus could have potential anti-diabetic effects. It is well acknowledged that drug treatment alone is frequently associated with negative side effects; a combination treatment of two or more compounds could achieve synergistic therapeutic effect, dose and toxicity reduction. Taking the current research and status into consideration, it could be hypothesized that combined drug and natural compound inhibitors of α-amylase and α-glucosidase could be very interesting due to their potential efficiency. More importantly, as a healthy diet is of high relevance to the management of type 2 diabetes, then there is a great necessity to investigate the combination of drugs and natural resource extracts. 

Green tea, which contains large quantity of polyphenols, is confirmed to have multiple healthy effects. The available literature indicates that green tea extracts [5,6], its polyphenols [7] and EGCG [8] lower the level of blood glucose and thus have anti-diabetic effects. It could be hypothesized that green tea, known as a popular daily beverage worldwide, may have useful interactions with other anti-diabetic drugs. Until recently little information is known about the interaction between acarbose, an effective first-line drug which has inhibitory activities against α-amylase and α-glucosidase, and green tea. The aim of this study is to determine whether green tea extracts (GTE), green tea polyphenols (GTP) or epigallocatechin gallate (EGCG) in combination with acarbose have additive, synergistic or antagonistic effects on the inhibition of α-amylase and α-glucosidase *in vitro* and to provide some insights on the intake of green tea, or its related extracts. Using a two-way experimental design, the effect of combinations of GTE, GTP, and EGCG and acarbose on α-glucosidase and α-amylase were studied and analyzed using the Chou and Talalay method, which is widely used to determine synergistic or antagonistic effects in combination studies.

## 2. Results and Discussion

### 2.1. Inhibition of Each Component Alone against α-Glucosidase and α-Amylase

The IC_50_ values of each sample and acarbose were calculated from the dose-response curves (not given in this paper). The results (Table 1) showed that the IC_50_ values of GTE, GTP and EGCG against α-glucosidase were 4.421 ± 0.018, 10.019 ± 0.017 and 5.272 ± 0.009, respectively, which were much lower than the IC_50_ of acarbose against α-glucosidase (4,822.783 ± 26.042) indicating that GTE, GTP, and EGCG strongly suppressed the α-glucosidase and that could possibly be utilized for the control of postprandial hyperglycemia. Though GTE, GTP, EGCG were efficient inhibitors of α-glucosidase, they exhibited poor inhibitory effects against α-amylase. Table 2 shows that the IC_50_ values against α-amylase of GTE, GTP and EGCG which were 4,020.157 ± 172.363, 1,370.812 ± 59.081 and 1,849.612 ± 73.475, respectively.

**Table 1:** The IC_50_ values of GTE, GTP, EGCG alone and their combination with acarbose on inhibiting α-glucosidase activity.

Components	IC_50_ (μg/mL)	
	Single	Combined
GTE	4.421 ± 0.018 *	2.104 ± 0.007 *
GTP	10.019 ± 0.017 *	3.999 ± 0.006 *
EGCG	5.272 ± 0.009 *	2.083 ± 0.004 *
Acarbose	4,822.783 ± 26.042	2,291.587 ± 13.014 *^†^ (GTE)
		1,925.614 ± 9.875 *^†^ (GTP)
		1,906.454 ± 6.892 *^†^ (EGCG)

* Indicates significant difference (*p* < 0.05) from the positive control of acarbose alone. ^†^ indicates IC_50_ of acarbose independent of other components (GTE, GTP, and EGCG respectively) in combined study. IC_50_: half maximal inhibitory concentration.

**Table 2:** The IC50 values of GTE, GTP, EGCG alone and their combination with acarbose on inhibiting α-amylase activity.

Components	IC_50_ (μg/mL)	
	Single	Combined
GTE	4,020.157 ± 172.363	2,845.987 ± 154.07
GTP	1,370.812 ± 59.081 *	609.463 ± 20.351 *
EGCG	1,849.612 ± 73.475 *	1,094.802 ± 55.992 *
		1,974.612 ± 18.653 *^†^ (GTE)
Acarbose	2,715.654 ± 24.709	1,206.974 ± 11.395 *^†^ (GTP)
		1,614.753 ± 14.691 *^†^ (EGCG)

* Indicates significant difference (*p* < 0.05) from the positive control of acarbose alone. ^†^ indicates IC_50_ of acarbose independent of other components (GTE, GTP, and EGCG respectively) in combined study.

### 2.2. Combined Effects of Each Sample and Acarbose against α-Amylase and α-Glucosidase

**Figure 1:**
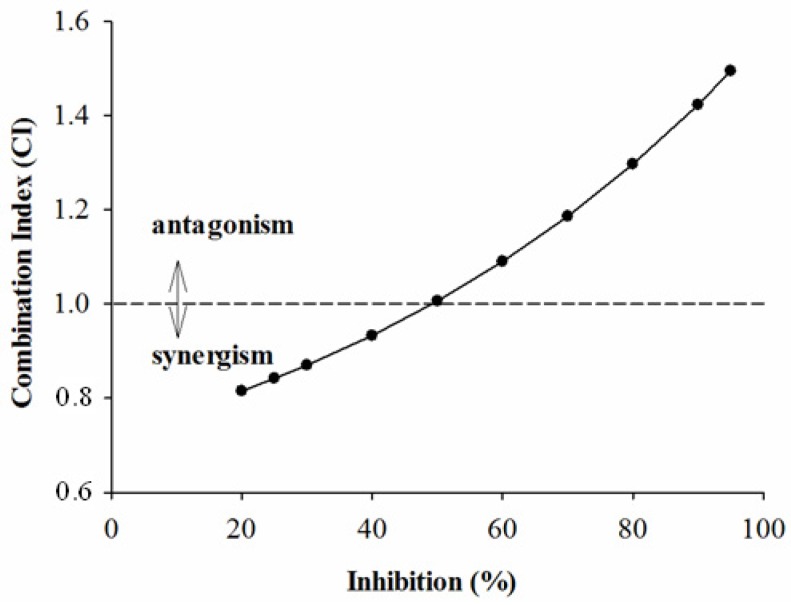
The CI of interaction between GTE and acarbose on α-glucosidase.

**Figure 2:**
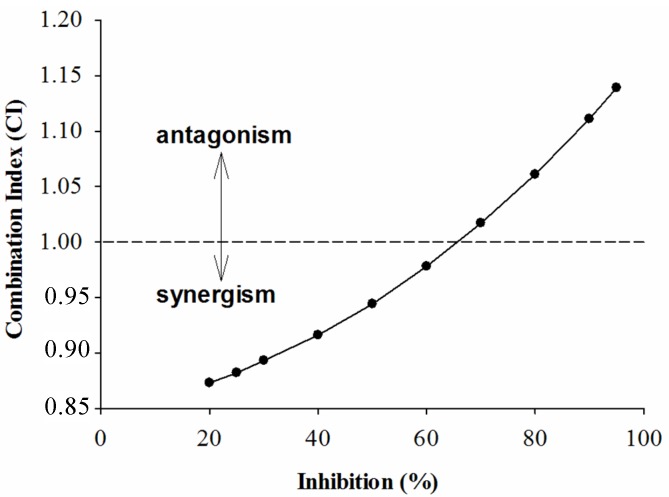
The CI of interaction between GTP and acarbose on α-glucosidase.

**Figure 3:**
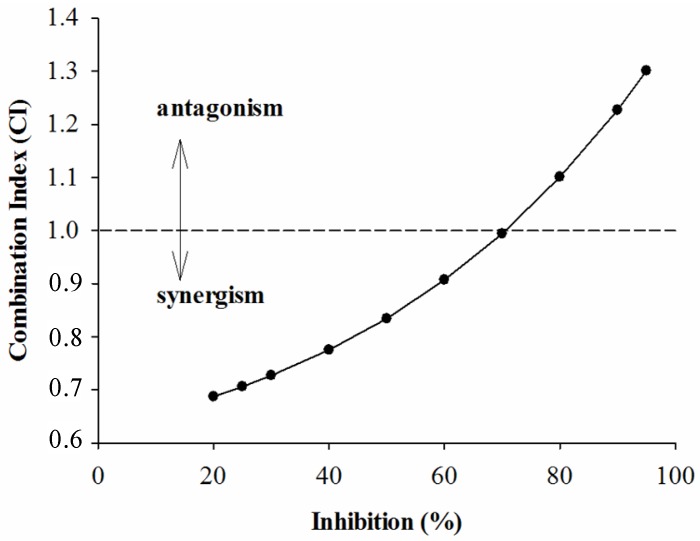
The CI of interaction between EGCG and acarbose on α-glucosidase.

In order to further investigate the combined effects of each sample and acarbose against the two enzymes, the plots of CI *versus* inhibition were generated and are given below. According to the CI values presented in Figure 1, Figure 2 and Figure 3, it is apparent that as expected, the combinations of GTE, GTP, EGCG and acarbose were complicated. Figure 1 shows that the combination of GTE and acarbose had a synergistic effect on α-glucosidase when the inhibition percentage was below 47%; at the inhibition percentage about 47%, the combination of GTE and acarbose was additive; however, the combined effect changed to antagonism at higher inhibition levels (above 47%). Similarly, it is noticed from Figure 2 and Figure 3 that the combination of GTP, EGCG and acarbose had similar synergistic effects when the inhibition percentages were below 62.5% and 68%, respectively. Based on the results, it can be concluded that the combination of GTE, GTP, EGCG and acarbose were synergistic at low concentration, whereas the combinations were antagonistic at high concentration. 

**Figure 4:**
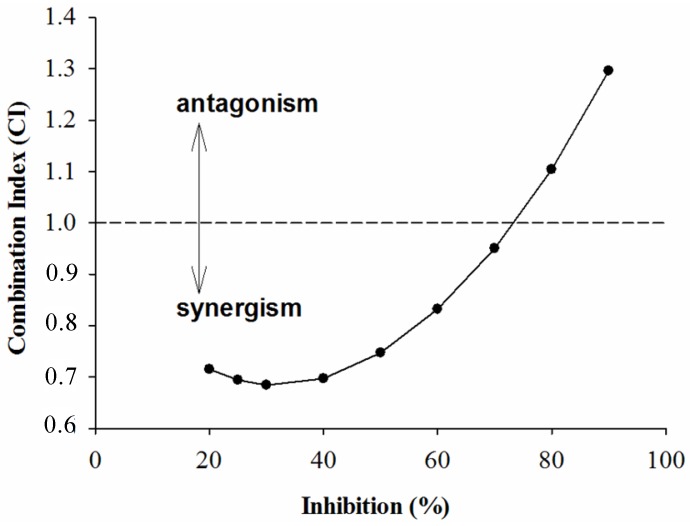
The CI of interaction between GTE and acarbose of inhibition on α-amylase.

**Figure 5:**
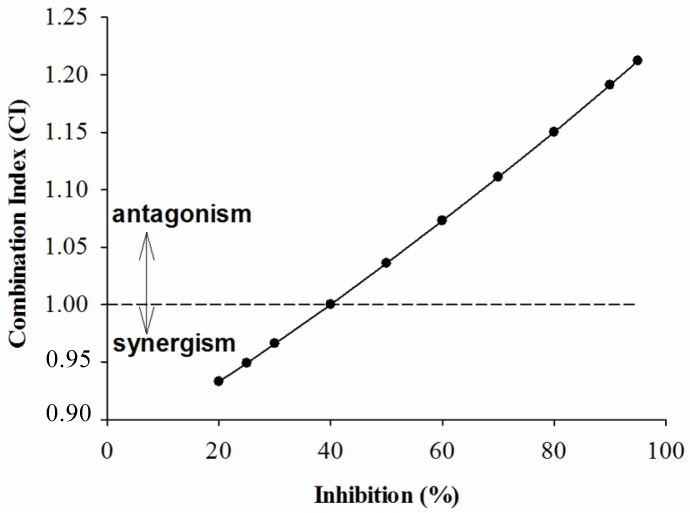
The CI of interaction between GTP and acarbose of inhibition on α-amylase.

**Figure 6:**
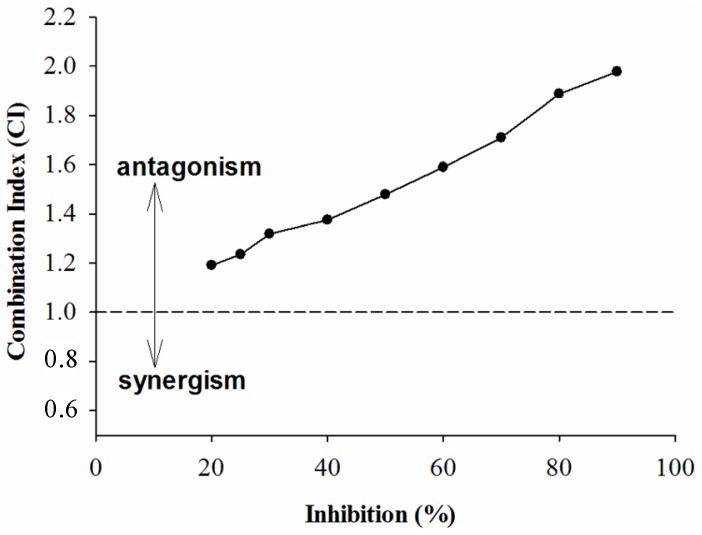
The CI of interaction between EGCG and acarbose of inhibition on α-amylase.

CI analysis of GTE, TP, EGCG and acarbose against α-amylase is shown in Figure 4, Figure 5, and Figure 6. It was seen from Figure 4 and Figure 5 that the CI value was equal to 1 at the inhibition percentages about 72% and 40%, respectively, suggesting that addition, synergism, and antagonism existed at the concentrations tested. Surprisingly, the combination of EGCG and acarbose on α-amylase were shown (Figure 6) to be antagonistic at the concentrations tested in our experiments. 

It is well established that the incidence of diabetic complications is reduced through the control of postprandial hyperglycemia. Inhibition of two main carbohydrate digestive enzymes, namely α-amylase and α-glucosidase, is currently regarded as an efficient way to manage postprandial hyperglycemia. Our present work confirms the fact that GTE, GTE, GTE, are all potent inhibitors of α-glucosidase, with 800–1000 times the efficacy of acarbose based on the IC_50_ values, whereas α-amylase is not strongly inhibited by them. Similar results were also reported in previous studies [9,10,11] and strong inhibitory activities against α-glucosidase could be considered as a possible mechanism for controlling type 2 diabetes.

It is possible that intake of green tea or functional food rich in tea polyphenols could be potentially regarded as at least a complementary therapy for postprandial hyperglycemia. It is also worth noting that GTE and EGCG exhibit higher inhibitory activities against α-glucosidase than GTP whereas the inhibitory activity between GTE and EGCG show no significant differences, which requires further exploration. 

A consensus has been developed that dietary intervention may be a practical approach to the improvement of the chemical therapy partly due to the naturally bioactive compounds in our daily food. Green tea, one of the most commonly consumed beverages in the World, is recognized in the prevention of diabetes. However, few papers report whether green tea or its polyphenols have interactions with diabetic drugs. Here we report for the first time the combined inhibitory effects of acarbose and GTE, GTP or EGCG against α-amylase and α-glucosidase *in vitro*. Our CI analysis indicated that the combination of acarbose and GTE or GTP or EGCG produce a synergistic effect at certain concentrations. Based on the IC_50_ values (Table 1 and Table 2) of the combination study, it is indicated that the concentrations of GTE, GTP, EGCG and acarbose decreased by nearly two-fold when equal inhibition (50%) was achieved. As a consequence of the synergistic effect, it could be inferred that intake of acarbose accompanied by a certain amount of green tea or healthy food containing GTP or EGCG may be more efficient than acarbose alone. Besides, it is well accepted that acarbose is linked to several side effects such as abdominal distention, flatulence, meteorism and diarrhea [12,13]. Then it could be expected that combined therapy with GTE or GTP or EGCG may diminish the dose of acarbose needed and the progressive increase in optimal drug dosage [14]. It should also be mentioned that the combined effect of acarbose and GTE or GTP or EGCG turned out to be antagonistic at higher concentrations in the combination study, which means the combined efficiency is diminished. Though our *in vitro* tests indicated that there was a synergistic interaction between the acarbose and GTE (or GTP or EGCG), *in vivo* studies should be conducted in order to evaluate the combined effect of acarbose and GTE (GTP or EGCG).

## 3. Experimental

Green tea water extracts (GTE), green tea polyphenols (GTP) and EGCG were purchased from Zhejiang Oriental Tea Corporation (Hangzhou, China) and their catechin components analyses are shown in Table 3. Acarbose, baker’s yeast α-glucosidase (EC 3.2.1.20) and porcine pancreatic α-amylase (EC 3.2.1.1) were purchased from Sigma Chemical Corporation (St. Louis, MO, USA). All the chemicals used were of analytical grade or chromatographic grade.

**Table 3:** Quantification of catechins in samples.

Samples	GA	GC	EGC	C	CAFF	EC	EGCG	GCG	ECG	CG
GTE	ND	10.89%	15.97%	1.66%	10.91%	11.75%	28.94%	1.51%	14.50%	3.84%
GTP	ND	ND	ND	1.07%	ND	8.75%	68.01%	4.91%	ND	16.67%
EGCG	ND	ND	ND	3.38%	ND	ND	95.62%	ND	ND	1.01%

GA, gallic acid; GC, (+)-gallocatechin; EGC, (−)-epigallocatechin; C, (+)-catechin; CAFF, caffeine;

EC, (−)-epicatechin; EGCG, (−)-epigallocatechin gallate; GCG, (+)-gallocatechin gallate;

ECG, (−)-epicatechin gallate; CG, (+)-catechin gallate; ND, not detected.

### 3.1. α-Glucosidase Inhibitory Activity

The α-glucosidase inhibitory activity was determined according to the method described in our previous study [15]. A mixture of sample (50 μL) and 0.1 M phosphate buffer (100 μL, pH 6.9) containing α-glucosidase solution (1U/mL) was incubated in 96 well plates at 25 °C for 10 min. After preincubation, 5 mM p-nitrophenyl-α-d-glucopyranoside (pNPG) solution in 0.1 M phosphate buffer (pH 6.9) (50 μL) was added to each well at timed intervals. The reaction mixtures were incubated at 25 °C for 5 min. Before and after incubation, absorbance was recorded at 405 nm by microplate reader (SpectraMax M5, Molecular Devices, Sunnyvale, CA, USA). The reference had 50 μL buffer solution in place of sample. The α-glucosidase inhibitory activity was expressed as inhibition percent and was calculated as follows:

Inhibition (%) = (ΔA_ref_ − ΔA_sam_)/ΔA_ref_ × 100 (1)

where A_ref_ is the absorbance of the reference; A_sam_ is the absorbance of the test samples.

### 3.2. α-Amylase Inhibition Activity

The α-amylase inhibitory activity of the tea fruit peel extracts was determined according to our previous study [15]. A total of 250 μL of sample and 0.02 M sodium phosphate buffer (125 μL, pH 6.9 with 6 mM NaCl) containing α-amylase solution (0.5 mg/mL) was incubated at 25 °C for 10 min. After preincubation, 1% starch solution in 0.02 M sodium phosphate buffer (pH 6.9 with 6 mM NaCl, 250 μL) was added to each tube at timed intervals. The reaction mixtures were then incubated at 25 °C for 10 min. The reaction was stopped with dinitrosalicylic acid color reagent (0.5 mL). The test tubes were then incubated in a boiling water bath for 5 min and cooled to room temperature. The reaction mixture was then diluted after adding distilled water (5 mL), and absorbance was measured at 540 nm. The reference had 50 μL buffer solutions in place of sample. The α-amylase inhibitory activity was calculated as follows:

Inhibition (%) = (A_ref_ − A_sam_)/A_ref_ × 100 (2)

where A_ref_ is the absorbance of the reference; A_sam_ is the absorbance of the test samples.

### 3.3. Experimental Design of Combination Study

To evaluate the effect of tea compounds (GTE, GTP, and EGCG) with acarbose, first, the IC_50_ value of GTE against α-glucosidase and α-amylase was calculated based on dose-response curves. Then a series of six concentrations of GTE (at 2.5IC_50_, 2IC_50_, 1.5IC_50_, IC_50_, 0.5IC_50_, 0.25IC_50_) were prepared and combined with equal concentrations acarbose at equal volume to be used to examine GTE and acarbose combined inhibitory effects on α-glucosidase and α-amylase. The same experimental design described above was used for GTP and acarbose and EGCG and acarbose solutions. 

### 3.4. Mathematical Analysis

In order to determine the addition, synergism, and antagonism of the combination of green tea extracts, green tea polyphenols, EGCG and acarbose against the two enzymes, the median-effect principle and combination index developed by Chou and Talalay [16,17] were introduced in this paper. The equation of median-effect principle is defined by:

log (f_a_/f_u_) = m log D − m log D_m_ (3)

where D is the dose, D_m_ is the dose required for 50% inhibition effect, f_a_ is the fraction affected by dose D, and m is a coefficient of the sigmoidicity of the dose-effect curve, f_u_ = 1 − f_a_.

The equation of combination index (CI) is expressed by:

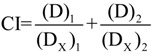
(4)
where (D)_1_ and (D)_2_ are the doses of samples and acarbose in the combination system, respectively; (D_x_)_1_ and (D_x_)_2_ are the doses of sample and acarbose alone, respectively. CI < 1, CI = 1, and CI > 1 indicate synergistic, additive, or antagonistic effects, respectively. Here synergism (or antagonism) does mean enhancement (or attenuation) based on the Chou-Talalay method for drug combination.

### 3.5. Statistic Analysis

All data were expressed as the mean ± SD of three replications of the experiment. Statistical analysis was performed by One-way analysis of variance (ANOVA) and Student’s t test. Significant difference was considered at *p* < 0.05.

## 4. Conclusions

The combination of GTE, GTP or EGCG with acarbose had a synergistic effect on α-amylase and α-glucosidase at low concentrations, though the combined effect turned to be antagonistic at high concentrations according to the CI values. These findings provide some valuable implications for the combination of acarbose and GTE (GTP or EGCG) in the treatment of diabetes mellitus that beg further examination.
